# Mémoire sur les Rétrécissements Organiques du Canal de L’Urètre et sur L’Emploi de Nouveaux Instruments de Sacrification, &c.

**Published:** 1843-07

**Authors:** 


### REVIEWS.

---

ART. II.—*Mémoire sur les Rétrécissements Organiques du Canal de l'Urètre et sur l'Emploi de Nouveaux Instruments de Scarification, &c.*

*Memoir on Organic Strictures of the Urethra and on the Employment of new Scarifying Instruments for the Radical Cure of that Disease; with plates.* By MARTIAL DUPIERRIS, M.D., of New-Orleans; 8vo. p. 120: Paris, 1840.

The work of Dr. Dupierris, without laying any claims to the character of a complete systematic treatise on organic strictures of the urethra, presents an excellent outline of the existing state of the science in relation to that important malady, with an account of a new set of scarifying instruments which appear to be well adapted to the object for which they are intended. During his residence in New-Orleans he devoted much attention to the study of this class of affections, and had ample opportunities of testing the value of the various modes of treatment pursued by surgeons. The first forty pages of the memoir are occupied with an account of the anatomy and physiology of the urethra, the nature, seat, causes, and diagnosis of strictures, and the manner of exploring that canal. The remainder is devoted to the discussion of the treatment of the disease, and to the recital of eleven cases in which the author successfully employed the method of scarification with the instruments of which he is the inventor, and to which we shall presently more particularly refer.

As Dr. Dupierris very justly remarks, there is still much contrariety of opinion respecting the seat of this affection. Hunter, who alledges that he has never witnessed it in the prostatic portion of the canal, observes that it is most commonly met with near the bulb, and similar statements have been made by Desault, Chopart, and Sœmmering. According to Ducamp the disease exists five times out of six at the distance of from four inches and a half to five and a half from the meatus urinarius. The same writer states that, in a very great number of patients, he has found the disease only twice at the external orifice of the canal, and in only two other cases two inches further down. Boyer does not admit the opinion of Bell, who says he has never met with stricture behind the internal "fascia" of the perinæum. The French surgeon declares that the obstruction ordinarily occupies the membranous portion of the tube, and that it is rarely observed below the penis. In twelve patients spoken of by Lallemand the disease existed at the curve of the urethra, or a little beyond it: in one case only did it occupy the straight portion of the canal. He concludes from an examination of the cases recorded by Ducamp that, in twenty-nine strictures found in sixteen individuals, eleven occurred at the distance of a little more than five inches and a half from the outer orifice, four at a little less than four inches and a half, and only fourteen at the place indicated by Ducamp. These facts, added to those derived from his own experience, lead him to believe that strictures are often seated at the neck of the bladder. Differing from this, again, is the opinion of Civiale, who has, perhaps, devoted more attention to the subject than any other writer of the present day. According to him, the only regions in which true organic strictures are found are, first, the external orifice of the canal; secondly, the two extremities of the navicular fossa; thirdly, the anterior part of the spongy portion; and fourthly, the sub-pubic curve at the junction of the bulbous and membranous divisions.

In nine cases in which the author carefully noted the distance of the disease from the external orifice, it was found in three between two and three inches; in three at about five inches; in two at six inches and a few lines; in one at three inches. These results, it will be observed, do not differ materially from those obtained by Civiale.

Mr. S. Cooper* thinks the disease most commonly occurs just behind the bulb of the urethra, the distance from the external orifice being six and a half or seven inches. The situation, next in order of frequency, is about four inches and a half from the orifice of the glans. Strictures, he says, do also form at three inches and a half from this point, and sometimes almost close to it. In some cases the external orifice itself is contracted. Mr. Hunter never met with the disease in the prostatic portion of the urethra. In almost all the cases observed by Sir Everard Home,† there was one stricture about seven inches from the mouth of the canal, whether there were others or not. Mr. Syme states‡ that the most common seat of the malady is at the bulb, about six inches from the extremity of the penis. The one next in point of frequency is at the part where the penis bends upon itself when pendulous, which is about three inches and a half from the orifice. The other situations particularly exposed to its occurrence are the neck of the glans and orifice of the urethra.

We have referred to some of the principal authorities upon this subject for the purpose merely of showing their contradictory character, not with the hope of throwing any useful light upon it. The fact is, organic strictures of the urethra may occur in any part of its extent anterior to the prostate gland; but the question as to whether they are more frequently seen at one point than at another can scarcely be said to be finally settled.

The diagnosis of strictures of the urethra can be satisfactorily determined only by a careful examination. This, the author thinks, should not be limited, as is too generally the

---

* First Lines of Surgery, vol. ii, p. 274.

† Practical Observations on Strictures.

‡ Principles of Surgery, third edition, p. 348.

case, to the internal surface of the canal, but should be extended to the exterior of the penis. By passing the thumb and forefinger lightly along the course of the urethra we may often derive important information not only in regard to the seat and number of strictures, but also in respect to their size and consistence. This is particularly true of the part of the canal anterior to the perinœum; behind this point this external exploration is more difficult.

Having briefly discussed the treatment of strictures by dilatation and cauterization, as practised by many of the surgeons of the present day, Dr. Dupierris comes to the subject of scarification, the part of the work with which we are more particularly interested. The instruments which he has devised for this operation, and which he thinks are entirely free from the objections that have been urged against those of Amussat, Stafford, Ricord, and others, cannot be fully understood without the aid of a drawing, but the following account will serve to convey some idea of them.

A silver tube, either curved or straight, from two and a half to three lines in diameter, and of the length of a common catheter, presents, about half an inch from its vesical extremity, an enlargement or knob, four lines in extent; this knob is so arranged that it may be taken off or removed at pleasure, and supports a narrow or constricted portion, about two-thirds of an inch long, blunt at the free extremity, and cleft at the side, for the accommodotion of what may be called the *stricture-blade.* This little instrument is nearly oval in its form, pointed at the free end, blunt at the other, sharp at the edges, and secured to a silver rod in the interior of the tube; this latter has a sort of button-shaped head, and by moving this back and forth the stricture-blade is made to project across the lateral cleft adverted to, and so carried through the seat of the disease. In the curved instrument the blade is fastened to the internal rod by means of a piece of chain, which is so constructed as to be solid in its entire extent.

Before the operation is undertaken it is frequently necessary to combat the inflammatory disposition of the urethra. In some persons, indeed, the sensibility of the canal is so exquisite that they cannot tolerate the slightest attempt at the introduction of a sound or bougie. In such cases the tepid bath is indicated, together with demulcent drinks, fomentations to the hypogastric region, and local or even general bleeding. Injections of a weak solution of nitrate of silver or of a watery infusion of opium or belladonna, occasionally answer a very good purpose; but an indispensable condition in all cases, is to accustom the urethra to the presence of small bougies, the size of which is to be gradually increased until the canal admits of complete distention. This preliminary treatment is to be conducted with great care; the bougie is to be introduced very gently, and no attempt is to be made to scarify the stricture until the parts are in a proper state.

When this object has been accomplished, the coarctotôme, for so the instrument is called, is oiled, and introduced in the same manner as a common catheter, sound, or bougie. If there is any difficulty in engaging the beak of the coarctotôme in the stricture, it may be easily surmounted by moving it in a lateral or circular direction, taking care at the same time to make gentle tractions on the penis. The resistance offered to the instrument, and the finger applied to the seat of the obstruction, will indicate whether the beak is in the proper situation or not. When this has been ascertained, the surgeon moves the internal rod of the coarctotôme forward and backwards, and thus divides the stricture longitudinally on one side. When the stricture involves the greater part of the canal, or its entire circumference, and it is deemed advisable to incise it at several points, it is only necessary to turn the instrument, which can be done without the trouble or inconvenience of withdrawing and re-introducing it.

The moment the incisions are completed the instrument is pushed from before backwards, in order to engage the knob or enlarged portion previously adverted to, in the part of the urethra beyond the seat of the obstacle. It is then withdrawn, and a leaden or pewter bougie substituted. This is allowed to remain in for two days, being removed only when the patient has occasion to void his urine, and replaced immediately afterwards. At the end of this time a bigger instrument is introduced, and in this manner the treatment is conducted until the canal is capable of receiving one of the largest size.

The operation is rarely attended with much pain, nor does it give rise to any but the slightest hemorrhage. When the stricture is not very large or firm, one or two scarifications are generally sufficient; but where the contrary obtains it may be necessary to repeat them as often as six or eight times, at intervals of as many days. The cure is indicated by the size of the stream of urine and the absence of local uneasiness. Very little after treatment is necessary beyond the employment of emollient injections. When suppuration ensues, and there is a considerable discharge of pus, which, however, does not often happen, a gum-elastic bougie, smeared with simple cerate, may occasionally be introduced, and repeated until the little wounds are cicatrized. Some inequality or roughness now and then remains at the scarified part, especially when the healing process has been tardy and imperfect; to remove this, the best remedy is cauterization, conducted in the same manner as when we wish to destroy a stricture in the first instance.

Most writers suppose that the cure of strictures by this operation is effected altogether on mechanical principles; but this is not the case. The little incisions made by the coarctotôme are, it is true, filled with plastic lymph, but none of this substance remains after the cicatrization is completed, while it is equally certain that the organized matter upon which the obstruction depended is in time entirely absorbed. The process, therefore, by which the cure is brought about is a vital, not a mechanical one.

We have thus endeavored to present a succinct analysis of the contents of this monograph, especially of that portion of it which relates to the seat of strictures, and to the mode of treatment by scarification. That this plan possesses decided advantages over those of dilatation and cauterization cannot, we think, be doubted; at all events, it is worthy of a fair trial, and those who feel an interest in the subject will do well to procure the work of Dr. Dupierris, and examine it for themselves. Meanwhile, we take pleasure in informing them that the able author is preparing a new edition, which will probably appear during the ensuing winter, enriched by additional observation and reading. Havana, where he now resides, must afford him an extensive field for the treatment of this class of affections, and we shall be happy to lay before our readers, from time to time, an account of his labors in this and other departments of surgery, to which, we believe, he is exclusively devoted. S. D. G.

